# Electronic Clinical Safety Reporting System: A Benefits Evaluation

**DOI:** 10.2196/medinform.3316

**Published:** 2014-06-11

**Authors:** Pamela Elliott, Desmond Martin, Doreen Neville

**Affiliations:** ^1^Elliott's EnterprisesSt John's, NLCanada; ^2^Eastern HealthDepartment of ResearchSt John's, NLCanada; ^3^Memorial UniversityOffice of the Provost and Vice-President (Academic)St John's, NLCanada

**Keywords:** electronic occurrence reporting, electronic clinical safety reporting, adverse event reporting in health care, evaluating electronic reporting systems in health care, health information technology evaluations

## Abstract

**Background:**

Eastern Health, a large health care organization in Newfoundland and Labrador (NL), started a staged implementation of an electronic occurrence reporting system (used interchangeably with “clinical safety reporting system”) in 2008, completing Phase One in 2009. The electronic clinical safety reporting system (CSRS) was designed to replace a paper-based system. The CSRS involves reporting on occurrences such as falls, safety/security issues, medication errors, treatment and procedural mishaps, medical equipment malfunctions, and close calls. The electronic system was purchased from a vendor in the United Kingdom that had implemented the system in the United Kingdom and other places, such as British Columbia. The main objective of the new system was to improve the reporting process with the goal of improving clinical safety. The project was funded jointly by Eastern Health and Canada Health Infoway.

**Objective:**

The objectives of the evaluation were to: (1) assess the CSRS on achieving its stated objectives (particularly, the benefits realized and lessons learned), and (2) identify contributions, if any, that can be made to the emerging field of electronic clinical safety reporting.

**Methods:**

The evaluation involved mixed methods, including extensive stakeholder participation, pre/post comparative study design, and triangulation of data where possible. The data were collected from several sources, such as project documentation, occurrence reporting records, stakeholder workshops, surveys, focus groups, and key informant interviews.

**Results:**

The findings provided evidence that frontline staff and managers support the CSRS, identifying both benefits and areas for improvement. Many benefits were realized, such as increases in the number of occurrences reported, in occurrences reported within 48 hours, in occurrences reported by staff other than registered nurses, in close calls reported, and improved timelines for notification. There was also user satisfaction with the tool regarding ease of use, accessibility, and consistency. The implementation process encountered challenges related to customizing the software and the development of the classification system for coding occurrences. This impacted on the ability of the managers to close-out files in a timely fashion. The issues that were identified, and suggestions for improvements to the form itself, were shared with the Project Team as soon as they were noted. Changes were made to the system before the rollout.

**Conclusions:**

There were many benefits realized from the new system that can contribute to improved clinical safety. The participants preferred the electronic system over the paper-based system. The lessons learned during the implementation process resulted in recommendations that informed the rollout of the system in Eastern Health, and in other health care organizations in the province of Newfoundland and Labrador. This study also informed the evaluation of other health organizations in the province, which was completed in 2013.

## Introduction

### The Risks of Health Care

Florence Nightingale once wrote, “it may seem a strange principle to enunciate as the very first requirement in a hospital that it should do the sick no harm” [1]. That was over a hundred and fifty years ago, and yet, today that requirement of “doing no harm” is still identified as an issue in the health system. While the health system has changed since that time, the “doing no harm” to patients is part of the patient safety agenda worldwide in health care.

Health care is provided in a high risk environment. In a report by The National Steering Committee on Patient Safety [2], which outlines a strategy for improving patient safety in Canadian health care, a brief description of that high risk environment is provided,

Health care is provided 24 hours a day, seven days a week. Dramatic advances in the diagnosis and treatment of disease have made care processes more complex; however, many organizations are hampered by outdated modes of communication, record keeping, employee training, and traditional hierarchical authority structures. The aging population, resource limitations, a critical shortage of qualified health professionals in a growing list of locations and specialties, and challenges created by mergers, and restructuring within health care organizations, are creating unequalled strain on the systems, thus, increasing the likelihood of adverse events, sometimes with lethal consequences.

The National Steering Committee on Patient Safety, [2], p 5

Patient safety has been defined in the Canadian Patient Safety Dictionary as “the reduction and mitigation of unsafe acts within the health care system, as well as through the use of best practices shown to lead to optimal patient outcomes” [3].

### Patient Safety in Hospitals

The issue of patient safety has gained an increasing profile in recent years, especially since the publication of *To Err Is Human* by the Institute of Medicine in 2000. The report estimated that between 44,000 and 98,000 Americans die each year from adverse events at a cost to the nation of US $8.5 to $19 billion annually [4]. Other countries, including the United Kingdom, Australia, and New Zealand have investigated the extent of the problem, and clearly shown that adverse events are a global patient safety concern [5-9]. Baker et al [5] conducted a detailed study of patient safety in Canada, and revealed that 7.5% of adult acute care patients in Canadian hospitals in the year 2000 experienced an adverse event, and 36.9% of these events were deemed to be preventable. The study estimated that between 9250 and 13,750 deaths from adverse events could have been prevented. Their study also looked at similar studies in other countries (United Kingdom, Australia, New Zealand, and the United States), and found that adverse event rates ranged from 2.9% to 16.6% of acute care admissions. They point out that one of the key steps in promoting patient safety is to have a reporting system that allows adverse events and near misses/close calls to be recorded so that health care workers can learn from them and implement corrective action plans.

The development of reporting systems for adverse events in health care can be traced back to the late 1970s. Since then, many countries have been implementing reporting systems and moving to electronic systems. However, countries such as the United Kingdom, Australia, Japan, and the United States are ahead of other countries, including Canada, particularly as it relates to national reporting systems [7,10]

### Eastern Health

Eastern Health is the largest integrated health organization in Atlantic Canada, serving a regional population of more than 290,000, and offering tertiary level and specialty services to a population of about 500,000 across the province of Newfoundland and Labrador. Eastern Health was formed in 2005 as a merger of seven organizations. The organization has approximately 12,000 staff members, and operates 27 institutional health service facilities and community health services in 30 communities. The services provided by Eastern Health cover a wide range of services in three sectors: (1) acute, (2) long term, and (3) community [11].

Clinical safety reporting is used interchangeably with occurrence reporting at Eastern Health, and refers to a process that facilitates the identification and monitoring of adverse events and incidents that occur during health care treatment or service and/or within health care facilities. The reporting system is used to report on occurrences such as falls, safety/security issues for patients, medication errors, treatment and procedural mishaps, and medical equipment malfunctions. This is consistent with the definition and approach outlined in the report of the provincial Task Force on Adverse Health Events [12]. An individual who is involved in an occurrence or witnesses an occurrence completes a report form and forwards it to the manager. The form captures information such as the patient name, patient record number, diagnosis, location of the incident, type of occurrence, time of occurrence, impact on patient, notification information, assessment information, physician assessment, and follow-up actions required. The form is only one part of the reporting system. The manager has the primary responsibility for ensuring the communication gets to the appropriate levels of authority and ensuring appropriate follow-up action. Depending on the complexity of the occurrence, and the follow-up actions required, the process can take from a few minutes to a few days, particularly if much consultation has to take place in determining the resolutions.

Early in the newly merged organization, Eastern Health recognized the need to improve and standardize it’s occurrence reporting processes. Each of the organizations involved in the merger had their own reporting processes, most of which were paper based. There were issues with the paper reporting systems, such as inconsistencies in what was being reported, different forms in use throughout the region, delays in notification to the Quality and Risk Management (QRM) Department, incomplete forms, and lack of feedback to employees about what was being done to address the clinical safety issues identified [12]. In an effort to improve the reporting system, Eastern Health submitted a proposal to Canada Health Infoway, seeking funding to implement an electronic occurrence reporting system. Canada Health Infoway is a national organization with the mandate for promoting the implementation of electronic records in the health system throughout the country. The proposal identified 13 objectives, all aimed at the ultimate goal of improving clinical safety.

Canada Health Infoway approved the funding to implement an electronic occurrence reporting system in late 2007, and the implementation began in 2008. The project required the selection of a vendor for the software applications for the Web-based system. The vendor chosen was based out of the United Kingdom, where many of its hospitals were using the system. Also, in Canada, the province of British Columbia (BC) had chosen the same vendor, and other health organizations were considering the same system. The software has the ability for organizations to customize some of their processes and terminology used in the occurrence reporting. The software chosen was anticipated to not only provide a user-friendly, confidential electronic form, but also help with other parts of the reporting process, such as the timely notification of the managers, improved communications between the different personnel involved, trending, and tracking.

Eastern Health is so large, that a staged implementation over several years was planned. The project budget included funding for a comprehensive evaluation of Phase One of the implementation, which involved four sites: (1) acute care, (2) long term care, (3) community health in an urban setting, and (4) an integrated services site in a rural setting. The evaluation was designed with the goal of determining if the anticipated benefits were realized, and if there were any lessons learned that could help with future implementations. The evaluation study was completed in 2010. The full evaluation was much more comprehensive in scope than presented in this paper. This paper outlines the evaluation approach used, and focuses on the key findings, particularly the benefits realized.

## Methods

### Evaluation of Health Information Technology

The evaluation of information technology (IT) in heath care is not conducive to the methods used in laboratory systems or “gold standards”, such as randomized controlled methods. Therefore, being able to identify causality is a limitation. A particular limitation is the difficulty in measuring or controlling for confounding variables, variables that are associated with the exposure of interest and also with the outcome of interest [13]. The physical settings, type of clinical service, acuity of patients, practices, and the experience of providers is not conducive to randomization and setting up control groups. Also, an important part of the evaluation of electronic health information systems is the end users’ acceptance of the system, and lessons learned which could assist in other implementations or system enhancements. Multi-methods, including the pre- and post-testing of interventions, is often advocated in health care IT evaluations. This quasi-experimental design is often used in the evaluation of health information systems due to time, cost, and technical restraints.

### Approach to This Evaluation

The approach to this evaluation was extensive, using both qualitative and quantitative methods. The design in this study involved measuring occurrence reporting data for a 6 month period before the implementation, and six months post implementation, as well as pre- and post-qualitative data. The design also involved a post test regarding user satisfaction, as well as the evaluation of training sessions.

The approach, including the development of data collection tools, was informed by five previous works in the evaluation of electronic health information systems and in patient safety. First, the work of Neville et al [14], which outlines a framework for evaluating electronic health records initiatives. The framework involves stakeholders throughout the process and utilizes a pre- and post-study design. Second, the work of Delone and McLean [15] on an information system success model, which has been incorporated by Canada Health Infoway into a benefits evaluation framework by Lau et al [16]. A key component of this framework involves the identification of indicators that can be used in the development of data collection tools to measure various dimensions of information systems success. Third, the work conducted by the British Columbia Electronic Incident Reporting Pilot Project in evaluating the same reporting system implemented at Eastern Health [17]. Fourth, the work of Ginsburg et al [18] and Accreditation Canada [19] in patient safety culture, and finally, pre-evaluation workshops attended by key stakeholders, which focused on the identification of research questions and indicators of interest.

The full evaluation study for the project focused on the following research questions, and used the data sources as outlined in Table 1. The scope of this paper is reporting on just a part of the larger evaluation, mainly on the benefits realized and the disadvantages/areas for improvement.

**Table 1:** Research questions and data sources used.

Research questions	Data sources
1) Anticipated benefits of this system.	Stakeholder workshops Project documents Literature review Focus groups Key informant interview
2) Benefits achieved and comparison with anticipated benefits.	Surveys Administrative records Focus groups Key informant interviews
3) Projected costs of this system.	Project documents
4) Costs of implementing the system and comparison with projected costs.	Project documents and discussion with implementation team
5) Necessary planning and management structures in place to proceed with the project.	Key informant interviews Focus groups Discussion with implementation team
6) Unforeseen harms and/or disadvantages.	Key informant interviews Focus groups
7) Key facilitators and barriers to successful implementation of the project.	Key informant interviews Focus groups Surveys Project documents

### The Indicators

The indicators chosen were based on the feedback that was obtained at a stakeholder workshop, and a review of the literature. Even though the full evaluation focused on many indicators, this paper will highlight the findings for the key indicators as follows: (1) number of occurrences reported, (2) reporter characteristics (nurses and non nurses), (3) timelines for reporting, (4) user satisfaction, (5) perceived benefits, and (6) perceived disadvantages.

The occurrence reporting data was compared 6 months post implementation to a similar 6 months period pre-implementation for each of the sites.

All of the frontline clinical staff and managers working in each of the four sites were included in the sampling for the user satisfaction questionnaires. These included staff such as registered nurses (RN), licensed practical nurses, personal care attendants, allied health professionals, ward clerks, diagnostic imaging, and laboratory staff. The physicians, research, and nondirect care staff were excluded from the sample. The rationale for the inclusions and exclusions was based on the historical utilization of occurrence reporting, and the planned implementation schedule. The user satisfaction survey questionnaire had close-ended questions, mainly about the electronic tool, and used a Likert-type scale.

The sampling for the interviews included all of the senior managers involved with the new system. The sampling for the focus groups included all of the frontline managers and frontline clinical staff at the sites who were using the new system. The pre- and post-focus groups and key informant guides used open-ended questions, focusing mostly on the perceived benefits and disadvantages/suggestions for improvement, as well as the facilitators and barriers.

## Results

### Response

Participation was voluntary for taking the satisfaction survey. There were 1074 user satisfaction surveys distributed post implementation, with 358 staff (330 frontline staff and 28 managers) responding for a response rate of 33.33%. Of the 358 who responded, 205 (57.3%) had used the system. The questionnaires were sent to the same staff pre- and post-implementation. Due to the nature of occurrence reporting, not all staff would have been involved in using the system during the study period, unless they had experienced or witnessed an occurrence. It is the staff that used the system that provided the data for the analysis related to the user satisfaction of the tool itself.

There were pre- and post-key informant interviews conducted, with 11 senior managers participating in both. There were pre- and post-focus groups conducted with the frontline managers and staff, with 12 managers and 13 frontline staff participating in the post implementation focus groups, as well as focused discussions with the project team. The qualitative results of all these groups and interviews contributed to the data discussed in this paper. A limitation is that there was low participation of frontline staff in the focus groups, even though the focus groups were held at lunchtime with lunch provided. Posters and email notices were provided at each site, but there was little response.

### Findings

In addition to the user satisfaction surveys, focus groups, and interviews, there was also a review of occurrence reporting administrative records for a 6 month period pre-implementation and 6 month post implementation. See Table 2, which compares the occurrence reporting data for a 6 month period before the implementation, to a 6 month period following the implementation.

**Table 2:** Comparison of the 6 months pre-implementation and the 6 months post implementation data.

Occurrence reports indicators	Pre-implementation (%)	Post-implementation (%)	Change/improvement between pre- and post-implementation (%)
# of occurrences reported	495	907	Increase 412 reports (83.2)
Reports completed	386 (77.9)	795 (87.6)	Increase (9.7)
Non-RN reports	129 (26.1)	391 (43.1)	Increase (17.0)
Reported within 48 hours of occurrence	166 (33.6)	799 (88.1)	Increase (54.5)
Average time between occurrence and notification of the manager sign-off	11.3 days	17 days	Increase 5.7 days (50.4)
Average time between occurrence and notification of quality and risk management	43 days	Immediately	Decrease 43 days (100.0)
Close calls	5 (1.0)	97 (10.7)	Increase (9.7)

### Key Benefits

The main findings of this study show that there are several key benefits realized, such as increased reporting of occurrences, improvement in the number of reports completed, more reporting by non-RN health care employees, improved notification of the managers and the QRM Department, and increased reporting of close calls. There were also some challenges experienced, such as decreased time in some areas for the close-out/sign-off of files. In addition to the changes in reporting, there was also satisfaction expressed by users with the new system.

The results of the user satisfaction surveys show that respondents across all care settings seem to be satisfied with the new electronic system. They report that the system is easy to use and consistent in performance. Other benefits identified in focus groups and interviews included: (1) easy access to computers and forms, (2) improved legibility, (3) increased awareness of what constitutes an occurrence and close call, (4) less time required to complete reports, (5) availability of information about the status of the individual managers’ occurrences, (6) easy to complete forms, (7) less paper shuffling, (8) more detailed information on reports, (9) easier to track follow-up actions, (10) improved confidentiality (reports not lying around at a nursing station for others to see), and (11) fewer misplaced reports. While all occurrence reports (paper or electronic) are expected to be confidential, paper reports are more vulnerable to being viewed by more than those involved in the occurrence. The electronic tool requires a password for access, and only those involved in completing the report, follow-up actions, and/or quality risk management personnel are permitted to view them.

### Areas for Improvement

Even though there were mostly positive comments about the reporting form, and most employees said they liked it, several areas for improvement were mentioned by the frontline staff. These included: (1) no place on the form for the person who attends to the client, the intervention, or a physician section to make notes; (2) form is too long; (3) locator drop down box does not lend itself to identifying the exact location of the occurrence (for example, “the room number”); and (4) the “locator function takes too long to scroll down to find the area of the occurrence”. Some participants also mentioned that sometimes there is not much feedback on the form from their managers regarding the follow-up action and prevention measures taken, however, they did indicate that they now have a reference file number for the report, and can see that it was reviewed.

## Discussion

### User Satisfaction

Many of the benefits identified are consistent with those identified in other studies. While the participants were not asked to prioritize or rank benefits, the ease of use was the most commonly mentioned. This is a similar finding to other studies with ease of use being the most frequently cited benefit [20-28]. Other benefits, such as those found in this study, are less cited. This study also included benefits not identified in the literature reviewed, such as the availability of information about the status of the individual manager’s occurrences, and fewer misplaced occurrence reports.

Even though many benefits were identified, there were several points of dissatisfaction raised by end users. For the management group, the inability to close-out files, and the uncertainty about whether or not the file was closed, were viewed as undesirable. When a report was changing handlers (a term used to describe the person following up on the report), they were unsure as to what happened with the report, as there was no confirmation if the handler received or acted on the report. There was also confusion at times with respect to management responsibility for a particular report when an occurrence involved two departments and one employee. This inability to “close the gap” was a concern, because the managers felt that despite the fact that they had taken appropriate follow-up action, it was not showing in the system in a timely fashion. There was also recognition that the implementation was not yet completed with respect to the coding classification of occurrences, and consequently, the managers were not able to get timely customized reports. At the time of this evaluation, work on these issues by the Project Implementation Team was in progress, and the managers indicated that addressing these functionality issues and getting the reports would enhance their view of the system. Although these issues were raised in the focus groups and interviews, the managers who responded to the user satisfaction survey expressed a high degree of satisfaction with the tool.

The issue of the locator function on the form itself was similar to a finding from a study on the same system by Walsh and Antony [29], where the location of the incidents was identified as a concern. The locator function is a feature that can be customized to the setting. The issue identified in the interviews and focus groups with the lack of customized “drop down boxes” was for specialized areas, such as laboratory and pharmacy services. The staff from the nursing areas, however, indicated satisfaction with the drop down boxes. The Project Implementation Team reported that there is a plan to customize the drop down boxes for the clinical support areas (eg, the Diagnostic Imaging, Laboratory, and Pharmacy Departments) to assist in making them user-friendlier for all end users.

As noted in the Findings section, the participants reported that there is no place on the form for employees to receive the feedback from their managers regarding the detailed follow-up actions and prevention measures taken. Other studies [24-26,30,31] point to the importance of feedback to staff, and that staff want to see that by taking the time to report an occurrence, there will be corrective action taken, and that quality will improve. It is well recognized that “you cannot fix what you cannot measure”. However, Clarke et al [30] point out that it is important to be aware of the types of problems that need to be fixed, rather than focus on all the instances of problems that need to be counted (p. 314). The counting can be used in tracking, but must be accompanied by action. The importance of receiving feedback on occurrences, and ensuring that corrective action is taken, was a common theme for both the managers and the frontline staff in this study.

### Changes in Reporting

There were notable increases in the numbers of occurrences reported in all settings, which is consistent with the findings from other studies [20,22,26,32]. While the number of occurrences increased across all sectors, it is difficult to analyze data about the types of occurrences across sectors. A review paper by Boxwala et al [33] examined various approaches to identifying errors and adverse events (of which occurrence reporting is one), and cautions about making any comparisons across sectors on the numbers and types of incidents, as there are factors such as inconsistent patient safety terminology, the clinical context including the roles of various personnel in the incident, the location, and other contributing factors.

A detailed breakdown of the types of occurrences reported by providers was not conducted. However, a high level review showed that there was a large increase (from 5 to 160) in the number of occurrences reported in the Clinical Assessment category. This category includes incomplete information on a requisition and/or specimen. This is consistent with the increase in reporting by the Diagnostic Services staff (radiology and laboratory), and was also mentioned in the focus groups and interviews. As in the pre-implementation period, the nurses were still the highest reporters for the Falls and Medications categories.

In a study by Zboril-Benson and Magee [34], there was an improvement in the types of incidents reported in a pilot project after cultural and educational changes were made. Pre-pilot reports at their study site indicated that only serious errors in health care were likely to be reported (ie, when a patient has been injured; when a willful violation of established protocol has been violated, etc). After the delivery of education sessions, they found an increase in the reporting of both close calls and occurrences with no harm.

The findings in the Zboril-Benson and Magee study are similar to the findings in this study. In the focus groups and key informant interviews, the participants indicated the education sessions that were conducted as part of the implementation process contributed to a better understanding and heightened awareness of the importance of reporting all occurrences and close calls. There was an increased awareness of what constitutes an occurrence. The participants indicated a better awareness of how the reporting of close calls can lead to system improvements.

An explanation given by a manager in this study, for an increase in reporting, was that even though all staff members in the paper-based system were expected to report occurrences (even when there was no harm to the patient), they did not, and often they just dealt with the issue. An example provided was that of a missing armband, “the staff would just do another armband for the patient and not write up the report”. The participants reported an increased understanding of how the tracking and trending of occurrences (even when there is no harm) can contribute to policy and practice changes.

Another contributing factor to the increase in reporting is the improved satisfaction expressed by employees with the ease of use and accessibility of the electronic tool. As was stated in the focus groups, “If staff members are busy, they may not bother to take the time from their day to find a paper report form and write up the occurrence, especially if no harm resulted to the patient”. The fact that the new system also provided a feedback mechanism to the reporter was identified as a benefit. Many reported that in the past, they completed reports, but often never heard back about what was done with the report or about the issues identified. As one participant described “it is like the report went in to the big black hole”. Now, it is easier to check on the status of the report, as they are given a file number when they log on and complete the electronic form.

### Reporter Characteristics

This study found a notable change in reporter characteristics post implementation of the electronic system, moving beyond the traditional RN reporter, from 129/495 (26.1%) to 391/907 (43.1%) of occurrences reported by other than RNs. Even though RNs were still the main reporting health care worker group, other workers, such as allied health professionals, ward clerks, medical records, diagnostic, and laboratory staff also reported more occurrences.

Nurses are still the most frequent reporters, and the most frequent types of occurrences are related to falls, medication administration, and safety/security issues in the clinical settings. This finding is consistent with those of previous research [22,26,35-37]. Blais et al [36] point out that because “nurses are often the professionals who fill out the incident report forms, the adverse events they report on are generally limited to the problems relevant to their work” (p. 11). Most other studies reviewed for this study focused on acute care. In all settings in this study (acute care, community, and long term care), nurses were still the predominant reporting category.

### Timelines for Reporting

There were improvements related to the timing of the notification of the occurrence to the QRM Department, and to the various management levels. In the past, the more serious occurrences, which resulted in harm to the patient, were usually reported as soon as possible, but often with occurrences that were of a lesser consequence, the reports were just sent over in the mail or notifications done when the manager could “get around to it”. The tool is designed to produce the immediate notification of the occurrence to the manager and the QRM Department, and can be customized for notification alerts to different managers, depending on the needs of the area. This immediate notification function was identified by the managers as one of the key benefits of the electronic system, as it improves the efficiency of the communication channels in the organization with respect to notification about occurrences. This finding is consistent with the Cochrane et al [22] study. The improved notification features also contributed to the increased number of occurrences reported within 48 hours of the occurrence. The increase in this study was 54.5% (88.1% -33.6% from Table 2 above) compared to the Cochrane study, which was 82%, the difference in the magnitude, being related to the difference in pre-implementation baseline timelines, where the Cochrane study was much lower on this indicator.  The post implementation timeline was similar for both studies, with 799/907 (88.1%) being the result in this study, and 84% being the result in the Cochrane study.

Post implementation, there was an increase in the average time (5.7 days) for the managers to sign-off on the report, compared to the previous paper-based reporting system, going from 11.3 to 17 days. The managers, quality leaders, and project leadership indicated that the decreased efficiency was related to the increase in the number of occurrences reported, as well as to the learning curve of the managers using the system. This new system resulted in an increased demand for follow-up activity, especially in areas where the number of occurrences had increased significantly, mainly in acute care, and the managers reported getting behind in completing files due to the other many competing demands on their time. The managers reported difficulties in understanding how to sign-off on the occurrence (follow-up completion), and they were not sure if they were completing this function correctly. As a result, the occurrence reports follow-up process, and closing-out the file were longer to complete overall. Hence, the system did not improve efficiency on this indicator during the 6 month post implementation period. This is in contrast to the study by Cochrane et al [22], where the average time between the event and the completion of the investigation decreased by 6 days, going from 39 days to 33 days. The baseline data was different. The time required for completing the investigation in BC (33 days) is longer than closing out a file in this study (17 days), but comparisons are difficult as the policies and procedures for closing out versus completing an investigation may differ, as well as the types of occurrences reported. The researchers in the Cochrane study [22] felt their result to be “only a slight improvement due to two factors: (1) the setting where the study took place was a busy unit where the manager had to support clinical work with limited opportunity to perform nonclinical, nonurgent work, which included doing follow-up work related to occurrence reports; and (2) the change in practice required of the manager was greater than anticipated” (p. 151). This was consistent with some of the feedback reported in this study. The managers reported that, in the past, they would “save up” the occurrence reports to complete them on “paper days”, when they could have uninterrupted time. The new system provides immediate notification; however, obtaining uninterrupted time in a busy setting to focus on the follow-up actions is a challenge. This did impact on the sign-off/close-out time, and thereby is being perceived as a disadvantage with the new system.

### Conclusions

This study showed that there are benefits to moving from a paper-based reporting of occurrences in health care to an electronic Web-based system. Some of the key benefits realized were an increase in the reporting of occurrences and close calls, improved timelines for notifying the managers and the QRM Department, improved tracking, more categories of the staff getting involved in reporting, reporting tool is easier to use, improved legibility, improved confidentiality, decreased reports missing, and more detailed information on the reports. It is important to point out that the implementation included extensive promotion and education of the new system, and this impacted on the awareness of employees, as identified in the interviews and focus groups. This, coupled with an easy to use electronic reporting tool, contributed to the benefits. The managers indicated that over time, the benefits realized would provide improved information that can lead to better tracking, trending, and addressing the clinical safety issues identified. While measuring the long term impact on clinical safety was beyond the scope of this evaluation, there was optimism expressed by the participants that if the employees continue to be engaged with the new system, then it will lead to improved clinical safety, as long as the issues identified are followed through with action plans. It would be interesting to repeat this study to see if the benefits realized after the first 6 months are sustained.

Most of the findings in this study are consistent with similar studies on Web-based electronic occurrence reporting systems in the acute care sector. The body of literature on the topic of benefits evaluations of electronic reporting systems in the acute care setting is small, and even smaller for the other sectors (community health and long term care). This study did include the long term care and community sectors, as well as the acute care settings, and the findings showed that there is little difference in the benefits realized between settings. The site was small for long term care (urban); therefore the findings from long term care settings have limitations. While some of the findings may be limited by the low participation of the frontline workers in the focus groups, the triangulation of the data from surveys, focus groups, interviews, and occurrence reporting records, provided evidence that there are benefits that can help in the pursuit of improved clinical safety, and that the employees support the system. The use of focus groups and key informant interviews provided information that was used to make improvements to the process and the tool.

The findings from this study were used to inform the rollout to the other sites at Eastern Health, and the implementation of the system in other health organizations in the province of Newfoundland and Labrador. There were changes made to the software and the implementation process based on the feedback obtained during the evaluation process. Also, the evaluation framework used in this study was used to guide the evaluation of the system in other regions in the province, which was completed in 2013. The evaluation approach for the provincial system used many of the same data collection tools as this study, but the amount of data collected was tailored to meet the resources available, as conducting pre- and post-studies can be quite costly. The evaluation tools and approach in this study have the potential to be used by other organizations that have the same or similar Web-based occurrence reporting systems.

## Abbreviations

BC: British Columbia
IT: information technology
QRM: quality and risk management
RN: registered nurse
